# Primary Sarcoma of the Tonsil: Removed by External Pharyngotomy1Read at the thirty-third annual meeting of the Medical Association of Central New York, held at Rochester, N. Y., October 16, 1900.

**Published:** 1901-01

**Authors:** Nathan Jacobson

**Affiliations:** Professor of clinical surgery, College of Medicine, Syracuse University; Surgeon to St. Joseph’s Hospital, Syracuse, N. Y.


### PRIMARY SARCOMA OF THE TONSIL: REMOVED BY EXTERNAL PHARYNGOTOMY.[1]

By NATHAN JACOBSON, M. D.,
Professor of clinical surgery, College of Medicine, Syracuse University; Surgeon to St. Joseph's Hospital, Syracuse, N. Y.

STEPHEN D., aged 31; Austrian; married. Referred to Dr. T. H. Halsted by Dr. J. F. Kauffman, May 1, 1900. During the past three or four years, on several occasions, had attacks of quinsy—relieved by incision. Eighteen months ago had what was again called quinsy. An opening was made, with temporary relief, but ever since there has been a sense of fulness in the throat, difficulty in swallowing, and a mass steadily increasing in size. Six months ago, while in Austria, the swelling was incised without benefit. General health has been good—only complaint being difficulty in swallowing and of late pain extending to left ear; voice thick and muffled; no dyspnea. Has lost some in flesh.

Examination showed left tonsil to be enormously enlarged, reaching down to and apparently involving the base of the tongue. Tumor extended across the median line, its vertical diameter estimated to be about three inches, the antero-posterior one and three-quarter inches, the transverse two inches. Tumor was adherent to the anterior and posterior pillars, but did not extend above the arch; nodular, hard as stone, but at some points less firm and, indeed, elastic, but no evidence of ulceration anywhere. Cervical lymphatic glands same side involved. No specific history nor evidence of specific disease.

Dr. Halsted made diagnosis of malignant disease of the tonsil and kept him under observation until June 21, 1900. During this time the growth slowly increased in size. Patient was given large doses of iodide of potassium. A small piece of the tumor, removed with cold snare, was sent to Dr. Kieffer for pathological study. He reported it to be nonmalignant. Dr. Halsted referred patient to me for operation. This was performed with the assistance of Drs. Halsted and Dayan at St. Joseph's Hospital, June 22, 1900. Before anesthesia was completed, cyanosis became so extreme that tracheotomy was imperatively demanded; venous bleeding very profuse, but ceased with introduction of the tracheotomy tube. Chloroform was used throughout the operation. The patient's head was held in a dependent position.

Incision was made along the inner border of the sternomastoid from a trifle back of the angle of the jaw and carried downward to the upper border of the thyroid cartilage. There was such a large

1. Read at the thirty-third annual meeting of the Medical Association of Central New York, held at Rochester, N. Y., October 16, 1900.

glandular mass in the submaxillary triangle, that to clear it out a second incision had to be carried upward over the lower jaw on a line with the angle of the mouth. Veins in this triangle were enormously enlarged. These were doubly ligated and severed between the ligatures.

After removing the diseased lymphatic glands I proceeded to expose the external carotid, and then tied its facial and lingual branches; cut my way through the muscles of the floor of the mouth, exposing the tumor. With the mouth held open with a gag, an assistant attempted to push the mass down to me by carrying his finger into the mouth. It was adherent to the soft palate, to the lateral wall of the buccal cavity, to the floor of the mouth and the lateral surface of the tongue, and extended downward into the neck, being attached to everything as far as the hyoid bone. It was impossible to save the hypoglossal nerve, as it was imbedded in the tumor. I cut away the stylohyoid and the other muscles of the upper hyoid group at their attachments to the hyoid bone. It was very evident that the tumor, on account of its size, could not be removed without section of the lower jaw. After extraction of the second molar tooth, the jaw was divided with the chain saw directly in front of the last molar. Drawing the severed ends of the inferior maxilla apart, I separated the tumor from the palate and other structures to which it was attached, by means of a scissors. A great many ligatures were required to control bleeding. Oozing continued, but was nicely arrested by suprarenal extract dusted into the wound. The jaw was secured by wiring without drilling, by carrying the wires around the neck of teeth. Closed the wound by interrupted silkworm-gut and catgut sutures and drained through the outer and upper part of my incision, using iodoform gauze wicking. During the latter part of the operation an intravenous saline injection seemed necessary. He was put to bed in good condition and stimulants were administered hypodermatically and per rectum.

For the first three days after operation his nourishment was administered entirely by rectum. Patient's progress towards recovery was interrupted by a mild pneumonia, which began immediately after operation. During this period the temperature rose to between 101° and 103°, and pulse and respiration correspondingly accelerated. A profuse expectoration of foul odor necessitated the continued use of the tracheotomy tube for nine days. The wound in the trachea healed kindly.

The wiring failed to hold the severed ends of the jaw satisfactorily, so Dr. Dayan took an impression of the jaw and made an interdental splint, which worked admirably, permitting him immediate use of his jaws. This was applied on the 7th of July.

His general health has steadily improved. He weighs some twenty-five pounds more than at the time of operation. There is a partial paralysis of the lower part of the face and of the left half of the tongue. There was some interference with the repair of the jaw. An infectious periostitis occurred at the point of section, with slight exfoliation of bone, which, however, is quite firm now, although it is still thought best to allow him to wear the splint, as it causes no discomfort and gives him firmer support.

Dr. Theo. L. Kieffer, who as stated made the first microscopic examination, made sections of the growth after its removal and pronounced it to be a small round-celled sarcoma.

Through the kindness of Mr. W. H. Walmsley, of Philadelphia, I am able to present to you three photomicrographs of 70, 95 and 170 diameters respectively. I present to you, furthermore, both the specimen removed and the patient operated upon.

### COMMENTS.

I shall not attempt an exhaustive consideration of this subject. Time does not permit it.

Malignant disease of the tonsil is rare. MacCoy records that of 10,000 malignant tumors treated in four London hospitals, but nine occurred in the tonsils, and of these nine but one proved to be a sarcoma. In 1887, Butlin stated in his work on "Operative Treatment of Malignant Disease," that he had been able to find records of but twenty-three cases operated upon for this condition. Newman, *Journal of American Medical Sciences*, May, 1892, reports that he has collected 144 cases, operation being attempted, however, in only 56 of them.

We encounter both sarcoma and epithelioma of the tonsil. I have met one case of epithelioma also. I operated upon him at his home in Otisco, N. Y., November 14, 1889, at the request of and with the assistance of his physician, Dr. F. W. Sears. He was a farmer, 61 years of age, and had increasing dysphagia for a year. His general health was good. At the time of operation the tonsil was the size of a small hen's egg and presented an ulcerated faucial surface. It did not involve surrounding structures; reached almost to uvula. Caused great difficulty in swallowing and impeded breathing when he assumed a recumbent posture. Two previous efforts had been made by others to remove the growth. On these occasions general anesthesia was attempted. Each time he strangled so that operation was discontinued. I injected cocaine, 4 per cent., into the tonsil. Then securing the tonsil with tenaculum, threw a piano wire loop about the tonsil, circumscribing the gland completely, and with snare screwed down the loop as tightly as possible. I next passed a platinum wire loop around the tonsil and to the outer side of the cold wire already applied, and tightened it up also. Connecting it with the galvano-cautery, I cut off the gland. There was no bleeding, and patient made a prompt recovery. Now, after eleven years, he still lives and enjoys good health; has not had the slightest evidence of recurrence. Sections of that tonsil were made and the diagnosis of epithelioma confirmed.

The first case presents points of interest from diagnostic, pathologic and operative standpoints. It was called chronic tonsillitis by many physicians. The elasticity of the growth was mistaken for fluctuation and it was incised a number of times. The continued growth, the invasion of surrounding structures, and its course remaining uninfluenced by treatment, led Dr. Halsted to the diagnosis of malignant tumor. The possibility of its being gummatous indicated the administration of iodide of potassium. Forty-grain doses, given three times daily for a month, had no effect.

The fallacy of depending upon the microscopic examination of small fragments secured from superficial areas of a tumor is apparent in this case. Dr. Kieffer found nothing to warrant the diagnosis of malignancy in the small piece wired off, but he very promptly discovered the sarcomatous character of the growth on examination of the tumor after removal. While ordinarily we have no lymphatic implication in sarcoma, the secondary glandular involvement with sarcoma of the tonsil is not unusual. This has been noted in many other cases. An unusual condition was the apparently cheesy condition of one of the glands. Macroscopically it appeared to be tubercular. The microscope revealed its malignancy.

The advisability and choice of operation are really the most important questions before us. Butlin, in the work referred to, summarises his views as follows: 1. The prospect of permanent relief by operation is small, if indeed there be any. 2. Operation through the open mouth is not dangerous, but in no case has there been a cure—in several, temporary relief. 3. Removal through external pharyngotomy is a dangerous proceeding—has not yielded as good results as through the mouth and cannot be recommended on future cases.

If this were true, the outlook would be gloomy, indeed. The experience of the profession since the publication of Butlin's work, thirteen years ago, fails to justify these conclusions. Newman has taken pains to follow up the operated cases, and in the article referred to gives us the benefit of his labors. There have been a number of permanent recoveries. Since the publication of his article, Park, McBurney and others have reported cases outliving a period of two or more years without recurrence.

My case of epithelioma of the tonsil disproves Butlin's statement that no malignant tumor removed by the mouth has been permanently cured. This operation, I believe, should be restricted to such cases as may be positively limited to the tonsil. Where other structures are invaded, where secondary glandular disease is present, external pharyngotomy is much more certain to be radical.

In a number of cases there has been no local recurrence, but the disease has appeared at some remote point and has resulted fatally. In at least two instances, singularly enough, the recurrence was in the opposite tonsil. In one of Newman's the second tonsil showed the presence of sarcoma years after the removal of the first. In one treated by Macintyre the second tonsil became involved eight months after removal of the first, without the slightest evidence of recurrence at site of first operation.

A few words as to external pharyngotomy: There is no doubt that it is an operation of seriousness and magnitude. The time allotted me will not permit of a consideration of the questions of technique. I will, however, call attention to a few points brought out by my case.

The extensive glandular implication required my making a rectangular incision. A number of large veins, seen usually with sarcoma, complicated and delayed the procedure. The invasion of the suprahyoid region greatly extended the areas of disease and correspondingly increased the difficulties of the operation. The extreme dyspnea when the patient assumed the recumbent posture, especially when the fauces were relaxed by the anesthetic, left no choice as to whether a preliminary tracheotomy was to be performed or not. It simply had to be done if we were to proceed. The venous hemorrhage for a few minutes was very profuse, but ceased spontaneously. With the head kept in the dependent position it was not necessary to tampon the larynx. For a long time I made every effort to save the hypoglossal nerve, but finally had to abandon the attempt. The paralysis of the suprahyoid muscles and those of the tongue supplied by it has not been sufficiently marked to cause particular trouble.

I wish especially to call attention to the interdental splint devised by our dental surgeon, Dr. Dayan. Another time I should have the impression of the jaw taken before operation and the splint placed at the time. In our case the infection of the jaw led to bone necrosis and the exfoliation of some bone. The contraction of the jaw in consequence necessitated the making of a second splint. While the union is quite firm, it is still thought wise to have him wear the splint for a short time.

Every effort will be made to keep our patient under observation, that we may know of the ultimate course of the disease in his case.

---

At the conclusion of his paper, Dr. JACOBSON made the following remarks:

I present this patient to you. You have here the line of incision. You have the mark here, where the tracheotomy tube entered and scarcely left a scar. I don't know whether you can see the line of incision there. The glands were removed from here. You see how large they were. You see he is still wearing that little piece of splint, because the fibrous tissue is not quite as strong as we would like to have it, but he can use his jaw and chew with this splint, which is put into position. Of course, I understand that four months after removal of a malignant tumor is by no means indicative of a cure, and we will watch his case; and now that a few cases have been recorded in which a cure has taken place and where they have outlived the period of three years, we hope this will be the case here. This is, of course, one of the malignant cases. I believe that we are getting experience enough to say that we are warranted in this procedure, and that cases will not be allowed to die unoperated, as was the case with our late President Grant. I think you will all be interested in this case. This man certainly does not present an unhealthy appearance for one who has undergone such an operation.

